# Structure-aware medical image fusion via mean curvature enhancement in the contourlet domain

**DOI:** 10.1371/journal.pone.0332869

**Published:** 2025-09-29

**Authors:** Shweta Sharma, Shalli Rani, Ayush Dogra, Mohammad Shabaz

**Affiliations:** 1 Chitkara University Institute of Engineering and Technology, Chitkara University, Rajpura, Punjab, India; 2 Marwadi University Research Center, Department of Computer Engineering, Faculty of Engineering and Technology, Marwadi University, Rajkot, 360003, Gujarat, India; Chengdu University of Traditional Chinese Medicine Wenjiang Campus: Chengdu University of Traditional Chinese Medicine, CHINA

## Abstract

The medical image fusion is a critical application in medical diagnosis, where anatomical and functional information from different imaging modalities, e.g., Magnetic Resonance Imaging (MRI) and Computed Tomography (CT) can be integrated. However, edge preservation, texture richness and structure consistency are a major challenge in complex fusion scenarios. This paper presents a novel multimodal medical image fusion technique based on the Contourlet Transform for multiscale directional decomposition and mean curvature filter for edge preservation. The proposed approach decomposes the source images into low- frequency and high-frequency components via a three-level Contourlet Transform. The low-frequency layers are fused via weighted averaging for brightness consistency, while the detail layers are processed by the mean curvature filter and then fused via maximum absolute selection to maintain edges and texture. The approach was evaluated against a variety of multimodal medical image datasets with consistent improvements against conventional methods such as Guided Filter Fusion (GFF), Laplacian Pyramid (LP), and Discrete Wavelet Transform (DWT). Experimental results showed average improvement of 19.4% in Spatial Frequency (SF), 17.6% in Average Gradient (AG), and 13.2% in Entropy (EN) over baseline methods. The results demonstrate that the method is useful for medical applications such as brain tumor localization, tissue differentiation, and surgery planning where high fidelity within fused imaging is critical.

## 1 Introduction

Medical imaging is the foundation of diagnosis, treatment planning and surgical guidance for modern healthcare. CT, MRI, Positron Emission Tomography (PET), Ultrasound (US) and Single-Photon Emission Computed Tomography (SPECT) are among the technologies providing different perspectives on anatomy, functional, and molecular behaviors of the human body. Each method is therefore limited in its ability to fully gather all significant information [1,2]. CT scans, for instance, have useful bone structure visualising detail but poor soft tissue contrast [3]. Conversely, MRI is ideal in soft tissue separation but fails to identify bone structures with extreme clarity [4]. Despite their insufficient anatomical detail, functional modalities like PET and SPECT are essential for monitoring metabolic activity. Consequently, clinicians may need to evaluate several images separately to obtain a comprehensive view, which can be challenging and prone to diagnostic uncertainty [5].

Medical image fusion has developed as a key method combining complementing features from several modalities into a single, more useful image to overcome these constraints. This approach guarantees that both anatomical and functional characteristics are preserved and shown concurrently, improves tissue contrast, and increases spatial resolution. By delivering richer visual representations, image fusion significantly supports decision-making, especially in scenarios such as tumor detection, neurodegenerative disease monitoring, vascular assessment, and image-guided interventions [2].

Traditionally, image fusion methods have fused data using statistical modeling, multi-scale transformations, and pixel-level operations. Although somewhat effective, these techniques usually experience issues in noise suppression, detail preservation and maintaining structural integrity across modalities with varied resolutions [3]. Recent advances in Artificial Intelligence (AI), particularly Deep Learning (DL), have transformed the domain of medical image fusion to solve these challenges. Data-driven models may now automatically learn spatial and semantic representations from large medical datasets, hence enabling more intelligent and adaptive fusion processes. Attention mechanisms, deep autoencoders, and Convolutional Neural Networks (CNNs) have all shown remarkable effectiveness in capturing hierarchical features ensuring the accuracy and consistency of the fused image [4].

Newer developments like diffusion models and Generative Adversarial Networks (GANs) have created new paradigms for image fusion in addition to more conventional DL architectures. These approaches offer efficient solutions for noise-aware reconstruction, simultaneous fusion and augmentation, and high-resolution fusion. These models now make it possible to combine tri-modal data, expanding the quality of fused outputs in medical practice [5].

Beyond the domain of DL, multimodal fusion is being more and more assisted by optimization-driven designs and invertible networks, which allow bidirectional mapping and lossless feature translation. These methods guarantee that fusion not only combines information but does so in a reversible and interpretable way, hence preserving the diagnostic value of source modalities [6]. Moreover, ML approaches are being used to develop disease-specific fusion strategies, where systems are trained to identify and preserve the most diagnostically relevant features for conditions such as cancer, cardiovascular disorders, and neurodegenerative diseases [7].

These advances are also accompanied by the expansion of smart image analysis systems where fusion is combined with downstream activities such as segmentation, classification and anomaly detection. Hierarchical multi-scale fusion networks have further strengthened the granularity of fused features, resulting in improved performance in classification and recognition tasks [8]. As a result, fused images are no longer confined to visual augmentation but are actively integrated into end-to-end diagnostic pipelines [9].

The path of medical image fusion research is toward explainability, real-time capabilities and personalization as one looks forward. Future systems are expected to be more context-aware, combining patient history, information, and feedback to dynamically drive the fusion process [10]. Additionally, fusion approaches will increasingly focus on adapting across imaging protocols, scanner types, and patient demographics, ensuring resilience and generalizability in real-world medical situations [11].

By means of the integration of thorough and diagnostically rich representations, medical image fusion helps to overcome the constraints of single imaging modalities. Medical image fusion is a revolutionary force in medical diagnostics with the inclusion of sophisticated DL, optimization algorithms and scalable structures, hence enabling more accurate, efficient, and individualized healthcare solutions.

The remainder of this paper is organized as follows: Sect 2 reviews related work, Sect 3 presents preliminaries, Sect 4 describes the preprocessing steps, Sect 5 details the proposed fusion methodology, Sect 6 introduces the evaluation metrics, Sect 7 outlines the experimental setup, Sect 8 reports and analyzes the results, Sect 9 presents the ablation study and Sect 10 concludes the paper with future research directions.

### 1.1 Motivation

Multimodal medical imaging, by merging structural and functional data from complementary modalities such as CT and MRI, is crucial for diagnosis. However, because of limitations in preserving directional attributes, edge details, and intricate anatomical textures, the effective integration of diverse modalities continues to pose challenges. Traditional wavelet-based methods lack the ability to capture geometrical structures effectively. The Nonsubsampled Contourlet Transform (NSCT), with its multiscale and multidirectional capabilities, has shown significant potential in medical image fusion. As demonstrated in earlier work, NSCT combined with advanced fusion rules such as spatial frequency or neural network models results in improved structural retention and contrast enhancement in fused outputs [12]. Motivated by these strengths, this study proposes a hybrid fusion framework that integrates Contourlet decomposition and Mean Curvature Filtering to generate informative and edge-preserving fused medical images.

### 1.2 Our contribution

The main findings of this study are described below:

1. By combining a multi-resolution Contourlet Transform with mean curvature-based filtering, a new medical image fusion approach is suggested to improve anatomical detail and visual contrast.
2. The approach utilizes three-level Contourlet decomposition to obtain low and high-frequency components to best extract intensity and texture information from the input modalities.
3. These low frequency sub-bands are combined with a weighted average strategy whereas a mean curvature filter is applied carefully to the high-frequency details of the third level and fused using maximum absolute rule.
4. When assessed on several medical imaging datasets, the suggested fusion technique shows greater performance, hence outperforming current fusion techniques in visual contrast in the combined images.

## 2 Related work

The following section discusses the work of various authors in the related field.

A DL-based approach for multimodal medical image fusion and categorization was proposed by Veeraiah et al. [13]. The objective was to enhance the accuracy of diagnosis by integrating fusion with disease classification. Despite the fact that the method was to utilize neural networks to extract and combine characteristics from multiple imaging modalities, the study was ultimately withdrawn due to methodological and repeatability concerns. This underscores the importance of comprehensive validation and open reporting in medical image fusion research. Robustness, dependability, and ethical research practice compliance should be the primary focus of future endeavors in this field.

Liu et al. [14] proposed a medical image fusion method based on convolutional neural networks. This approach learns feature mappings directly from the source modalities and demonstrated promising fusion quality in early deep learning research. However, its performance heavily depends on large datasets and network design parameters.

Agrawal et al. [15] introduced a simplified parameter-adaptive DCPCNN model. Their method balances complexity with performance by reducing the number of trainable parameters, making it more suitable for real-time or embedded fusion tasks.

Lin et al. [16] developed a multibranch, multiscale neural network utilizing semantic perception for the fusion of multimodal medical images. This method divides the fusion process into multiple branches across various scales to capture both fine and coarse features. A semantic module enables the network to focus on diagnostically relevant regions, hence improving the clarity and precision of the integrated image. This approach’s benefit is its preservation of structure and improvement of textural richness. The model is complex and may require for significant computing resources. Future studies might emphasize maximizing the design while ensuring the fusion’s quality is preserved.

Sinha et al. [17] suggested a multi-modal fusion method employing an improved dual-channel pulse-coupled neural network (PCNN). The method uses two parallel PCNNs to extract and synchronize features from the source images, hence improving both spatial and spectral information retention. The method reduces data loss and maintains edge information well. This model’s ability to fit the firing activity of neurons across all modalities is one of its key benefits. Conversely, PCNN-based methods can be costly in terms of computation and require exact parameter tuning. Future studies can focus on maximizing the dual-channel architecture for time-sensitive applications.

Zhang et al. [18] proposed a unified dictionary joint sparse model for the integration of medical images. This approach ensures the successful extraction and integration of common features by developing a shared language across modalities. It retains the essential information from both images while eliminating redundancy. The approach yields distinct textures and contours in the resultant composition. A disadvantage is that sparse coding may incur significant computational costs. Future advancements may involve the formulation of expedited sparse approximation methodologies or the integration of this model into deep learning processes.

Jie et al. [19] introduced a multi-modality fusion approach using fuzzy set theory and compensation dictionary learning. This method balances noise suppression with detailed feature preservation and offers flexibility across imaging domains. However, it is sensitive to dictionary construction and may require tuning for medical applications.

Tang et al. [20] introduced MATR, a transformer-based approach for the fusion of multimodal medical images. The method utilizes multiscale adaptive transformers to capture long-range dependencies and cross-modal interactions. The model improves structural alignment and maintains intricate textures by the use of attention processes at various sizes. The primary advantage of this method is its strong contextual modeling, which surpasses most traditional convolutional techniques. Nevertheless, transformer models generally necessitate prolonged training periods and considerable training datasets. Future study may concentrate on hybrid models or adaptations of lightweight transformers to enhance the efficiency of medical imaging.

Bavirisetti et al. [21] combined MRI and CT image using guided image filter and image statistics. The technique keeps important structure information from both source images by using guided filtering to keep the edges and statistical features to drive the fusion process. The main advantage about this method is that it keeps the clarity and contrast of the output, which is very important for medical analysis. But the fact that it depends on the accuracy of the statistical readings could be a problem because it can’t be used in all imaging situations. In the future, researchers may focus on finding the best ways to use the statistical selection process so that it works better with more datasets.

Bavirisetti et al. [22] suggested a two-scale image fusion technique for combining visible and infrared images using saliency detection. Saliency maps are used to guide the fusion of the base and detail layers generated by the approach, which ensures that key features are adequately represented in the final image. The advantage of this approach is that it prioritizes important image regions, focusing on human visual perception. On the other hand, its performance could suffer in low contrast environments when important components are not well defined. Trial results demonstrated that the method enhances visual targets and eliminates unnecessary information. Adding adaptive saliency models to the system could improve the results of future research.

Liu et al. [23] presented a thorough framework for image fusion that combines sparse representation and multi-scale transformations. This technique uses the sparsity of signal representations in transform domains, including wavelet and contourlet transforms, to enable effective feature extraction and integration. The main benefit of this method is that it can be applied to a wide range of image data formats, including multi-modal and multi-focus images. It reduces data loss and guarantees spatial consistency. However, the approach requires significant computer resources for transform domain operations and sparse coding. Across several datasets, their experimental results showed excellent fusion quality. Future research may focus on real-time applications and computing efficiency optimization efficiency and explore real-time implementations.

Zhu et al. [24] proposed a method for medical image fusion based on phase congruency and local Laplacian energy in the NSCT domain. The approach achieves high visual clarity by enhancing perceptual features and improving contrast but suffers from redundancy introduced by NSCT, which may impact scalability.

Kumar et al. [25] proposed method based on pixel significance using the cross bilateral filter for image fusion. By combining images utilizing the edge-preserving features and calculating the importance of each pixel, this method maintains sharp edges and reduces noise. The main advantage of this method is its ability to enhance structural features, particularly those close to object edges. However, the method may not scale well for high-resolution images or real-time applications because it operates at the pixel level. The results demonstrated that this approach effectively strikes a balance between detail retention and noise reduction. Future research might incorporate hierarchical or multi-resolution methods to improve performance and scalability.

Li et al. [26] devised a fusion technique based on guided filtering to enhance edge information and minimize artifacts in the fused image. Guided filtering offers rapid, edge-aware smoothing with minimal computational demand, making it suitable for high-quality fusion. This method’s notable advantage is its ability to handle many types of visual content with consistent performance. Conversely, it may be less effective in instances where input images exhibit significant modality discrepancies, resulting in the loss of complementary information. Their research confirmed that the method yields structurally coherent fused images. Future directions may prioritize adaptive filtering methods to more effectively address modality variance.

Kurban [27] proposed a lightweight and general-purpose fusion strategy called Gaussian of Differences (GoD). It simplifies the image fusion process by applying statistical Gaussian differences to identify and merge significant features, demonstrating competitive accuracy with high efficiency and low computational demand.

Song et al. [28] introduced D2-LRR, a dual-decomposed MDLatLRR-based fusion method. This technique shows strong performance in preserving local texture and enhancing salient features but can sometimes exaggerate contrast, making it prone to over-enhancement.

Kumar et al. [29] developed a fusion technique for multi-focus and multispectral images by integrating the importance of the pixels with the discrete cosine harmonic wavelet transform. The method emphasizes critical pixel regions and employs frequency domain decomposition to enhance focus and spectral fidelity. The primary advantage of this technology is its ability to preserve spectral and spatial information while minimizing noise. Its susceptibility to variations in illumination and ambient noise in the source images, however, may constitute a disadvantage. The results indicated improvements in both visual quality and quantitative fusion metrics. Future research may explore hybrid decomposition methodologies or adaptive thresholding to enhance robustness. Punjabi et al. [30] proposed a deep learning-based framework for the classification of Alzheimer’s disease by combining MRI and PET neuroimaging modalities using convolutional neural networks (CNN). The study showed that modality fusion significantly improved the classification performance compared to using single modalities. Specifically, the fused features extracted from CNN enhanced the model’s ability to distinguish between the normal control, mild cognitive impairment, and Alzheimer’s disease groups. Wu et al. [31] presented a fusion technique for infrared and visible images that combines nonsubsampled contourlet transform (NSCT) with a pulse-coupled neural network (PCNN). The method utilizes dual-channel NSCT to decompose source images and PCNN for coefficient fusion, achieving better detail preservation and contrast enhancement in the fused image. Although primarily aimed at general imaging, the fusion principles are applicable to medical scenarios involving thermal and visual information. Ogbuanya et al. [32] developed a hybrid optimization strategy for the fusion of multimodal medical images. The proposed approach integrated a hybrid of particle swarm optimization and artificial bee colony algorithms to optimize the fusion rules. The method demonstrated improved computational efficiency and fusion quality in various pairs of medical images, including magnetic resonance imaging, CT, and PET scans. Guo et al. [33] introduced a trimodality fusion technique to enhance target delineation in brain tumor radiotherapy. The study used MRI, CT, and PET images to create a complete representation of tumor regions. Their fusion method effectively combined anatomical and metabolic information, thus supporting a more accurate radiotherapy planning. Niroshana et al. [34] proposed a fused image-based method to detect obstructive sleep apnea using a single-lead ECG signal. The ECG was transformed into fused images that were then analyzed using a 2D CNN. The model achieved a high classification performance, indicating that image-based representation of bio signals can be effective for diagnosing sleep disorders. Jamil et al. [35] proposed a precancerous change detection method for mammography images that combines mean ratio and log ratio features with fuzzy c-means classification, using Gabor filters for texture enhancement. Their approach effectively detects subtle tissue changes indicative of early breast cancer, highlighting the role of hybrid feature extraction and clustering in medical imaging.

Javed et al. [36] presented a rare case study of Bing–Neel Syndrome, a central nervous system manifestation of Waldenström macroglobulinemia, which clinically mimicked giant cell arteritis. The report underscores the importance of accurate imaging interpretation and differential diagnosis in complex pathological presentations.

Abdullah et al. [37] introduced a joint learning framework for fake news detection that integrates multiple feature modalities within a unified model. Although developed for text-based misinformation detection, the architecture demonstrates the broader applicability of multi-feature fusion strategies, which can inspire cross-domain applications in medical image analysis.

Different multi-modal medical image fusion techniques have been attempted, ranging from DL and transformer models to traditional filtering and sparse coding. They all have their strengths, e.g., edge preservation, semantic highlight, or efficiency, and their trade-offs, e.g., high resource demands or image condition sensitivity. PCNN, guided filtering, and pixel-significance-based methods are robust in structural definition and visual enhancement.

A concise overview of commonly used fusion methods, including their datasets, key contributions, and limitations, is provided in Table 1.

**Table 1:** Summary of existing medical image fusion techniques.

Ref.	Method	Dataset Used	Key Contributions	Limitations
[14]	CNN	AANLIB	Learns deep representations and adaptive fusion rules from data	Requires large training datasets and complex model design
[22]	Two-Scale	Infrared + Visible	Saliency-guided fusion of base and detail layers	Reduced performance in low-contrast scenarios
[23]	LP	AANLIB	Simple pyramid decomposition preserving both low- and high-frequency components	May suppress fine features and edges
[24]	NSCT	AANLIB	Directional multi-scale transform with spatial frequency-based fusion	High computational cost; transform redundancy
[25]	CBF	http://www.imagefusion.org/	Edge-preserving fusion using pixel significance	Sensitive to edge misalignment; lacks deep semantic guidance
[26]	GFF	Multi-modal datasets	Fast edge-aware fusion with low complexity	Less effective under modality intensity variation
[29]	DCHWT	http://www.imagefusion.org/	Multiscale harmonic wavelet decomposition, good detail preservation	May amplify noise; not adaptive to different image types

## 3 Preliminaries

### 3.1 Contourlet transform

Contourlet Transform is a new form of representing images. It is designed to solve the problems of traditional wavelet transforms, which can not capture the direction and shape in images. Unlike wavelets that can only choose a few directions, the Contourlet Transform forms a more effective means of displaying smooth edges and lines through its capacity to decompose images at multiple directions and scales [38].

#### 3.1.1 Double filter bank structure.

**Fig 1:**
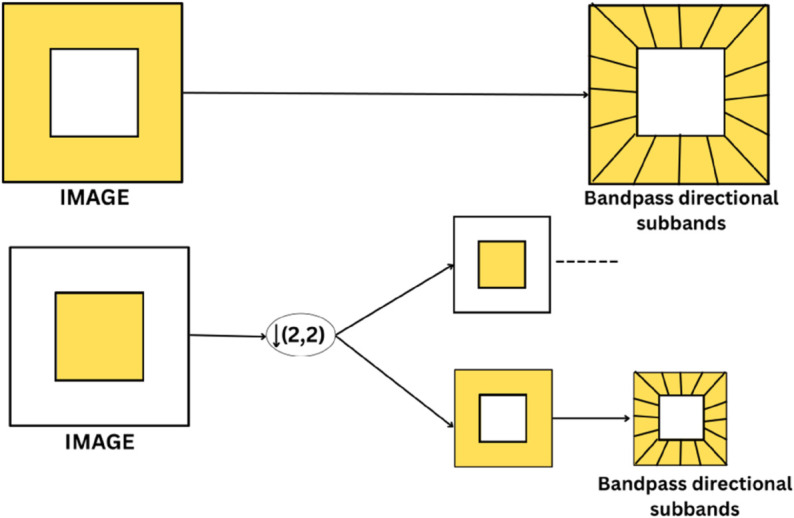
Contourlet filter bank: Multiscale decomposition via LP followed by directional decomposition using DFB [39].

The Contourlet Transform consists of a two-stage filtering process: a multiscale decomposition using a Laplacian Pyramid (LP) followed by a directional decomposition using a Directional Filter Bank (DFB) as shown in Fig 1 [39]. The LP is used to capture the point discontinuities (i.e., edges) in the image and decompose it into low-pass and band-pass subbands. The DFB then links the point discontinuities into linear structures by grouping frequency content in specific directions.

Let *f*(*x*,*y*) be the original grayscale image. The LP decomposition is expressed as:

$$L_{0}=f(x,y)$$ (1)

$$L_{i+1}=\text{Downsample}\left(L_{i}*h\right),\;i=0,1,\ldots ,n-1$$ (2)

$$H_{i}=L_{i}-\text{Upsample}\left(L_{i+1}*h\right),\;i=0,1,\ldots ,n-1$$ (3)

Here, *h* is a low-pass analysis filter, *L*_*i*_ is the low-frequency approximation at level *i*, and *H*_*i*_ is the corresponding band-pass detail component.

#### 3.1.2 Directional decomposition.

Each high-pass detail component *H*_*i*_ is further decomposed directionally using the DFB. This stage more precisely represents edge information in several directions by splitting the frequency spectrum into wedge-shaped directional subbands. One way to express the directional decomposition is as follows:

$$\left\{D_{i,j}{\}}_{j=1}^{2^{k}}=\text{DFB}(H_{i}\right)$$ (4)

where *D*_*i*,*j*_ is the directional subband at scale *i* and direction *j*, and 2^*k*^ is the number of directional channels at that scale.

#### 3.1.3 Reconstruction.

The reconstruction of the original image from Contourlet coefficients is achieved by first applying the inverse DFB on the directional subbands to reconstruct the band-pass component, followed by the inverse LP to synthesize the image across scales. The inverse LP can be written as:

$${\hat{L}}_{i}=\text{Upsample}\left(L_{i+1}*h\right)+H_{i}$$ (5)

The final reconstruction is given by:

$$\hat{f}(x,y)={\hat{L}}_{0}$$ (6)

The transform guarantees perfect reconstruction under ideal filter conditions, making it suitable for both analysis and synthesis tasks.

### 3.2 Curvature filter

Designed to enhance visual structures while reducing noise and variational artifacts, curvature filters are advanced edge-preserving smoothing techniques. These filters operate on the geometric properties of the image surface, regarding intensity values as a two-dimensional manifold lying within a higher-dimensional space. Emphasizing Total Variation (TV), Mean Curvature (MC), and Gaussian Curvature (GC) filters, this part describes the theoretical foundation and mathematical formulations of curvature filters.

#### 3.2.1 Total Variation (TV) filtering.

Total variation filtering minimizes the overall variation in the image, promoting piecewise smoothness and edge preservation [40]. The total variation of an image *u*(*x*,*y*) is given by:

$$TV(u)=\int_{\Omega }|\nabla u(x,y)|\;dxdy,$$ (7)

where ∇u denotes the image gradient and Ω is the image domain. The minimization of this functional leads to the well-known Rudin–Osher–Fatemi (ROF) model. The corresponding Euler–Lagrange equation governing the gradient descent evolution is:

$$\frac{\partial u}{\partial t}=\nabla \cdot \left(\frac{\nabla u}{\left|\nabla u\right|}\right).$$ (8)

This equation acts as a local curvature-based diffusion, smoothing the image in homogeneous regions while preserving sharp edges [41,42].

#### 3.2.2 Gaussian Curvature (GC) filtering.

Gaussian curvature takes into account the intrinsic geometry of the surface formed by the image intensity. The Gaussian curvature at a point is the product of the principal curvatures *k*_1_ and *k*_2_, and for a 2D image *u*(*x*,*y*), it is expressed as:

$$K(u)=\frac{u_{xx}u_{yy}-u_{xy}^{2}}{(1+u_{x}^{2}+u_{y}^{2}{)}^{2}},$$ (9)

where $u_{x},u_{y}$ are first-order partial derivatives, and $u_{xx},u_{yy},u_{xy}$ are second-order partial derivatives of *u*. Gaussian curvature filtering introduces anisotropic smoothing by distinguishing between ridge and saddle-like features and is particularly effective for preserving fine structures [43].

#### 3.2.3 Weighted mean curvature.

An enhanced version of MC filtering incorporates weights to adjust diffusion strength locally. The weighted mean curvature (WMC) is defined as:

$$\frac{\partial u}{\partial t}=-w(x,y)H(u),$$ (10)

where *w*(*x*,*y*) is a spatially varying function that controls the degree of smoothing based on local features [44].

These properties make curvature filters suitable for medical image fusion and other applications requiring structure-preserving enhancement.

### 3.3 Weighted averaging

Weighted averaging is a fundamental technique employed in pixel-level image fusion, particularly effective in the context of low-frequency component combination. It provides a straightforward yet reliable way to preserve the brightness and intensity consistency of the input images. This method is widely utilized due to its simplicity, computational efficiency, and ability to retain the overall visual structure of the source images.

Let *I*_1_(*x*,*y*) and *I*_2_(*x*,*y*) denote two spatially registered source images representing different imaging modalities, such as Magnetic Resonance Imaging (MRI) and Computed Tomography (CT). The fused image *F*(*x*,*y*) at pixel location (*x*,*y*) using weighted averaging is defined as:

$$F(x,y)=\alpha \cdot I_{1}(x,y)+(1-\alpha )\cdot I_{2}(x,y),$$ (11)

where α∈[0,1] is the weighting coefficient assigned to *I*_1_(*x*,*y*), and (1−α) is the weight assigned to *I*_2_(*x*,*y*). A common choice for *α* is 0.5, which ensures equal contribution from both modalities in the absence of any prior preference. This static or fixed weighting strategy is particularly effective when the goal is to combine the overall intensity features of both input images without introducing bias.

In the context of medical image fusion, particularly in low-frequency subband fusion derived from multiscale transforms like wavelets or contourlets, weighted averaging is used to maintain smooth intensity transitions and global contrast. Although it is effective in preserving average luminance, it may underperform in retaining edge features or fine textures when the source modalities differ substantially in information content. Therefore, it is commonly complemented with more sophisticated rules for high-frequency fusion, such as maximum selection or sparse representations [45].

### 3.4 Maximum-absolute selection rule

The Maximum Absolute Selection Rule (MASR) is a commonly adopted decision-level fusion strategy used for combining high-frequency components of source images in multiscale transform domains. This method is especially suitable in medical image fusion where sharp anatomical details and edges must be preserved from multiple imaging modalities. As introduced by Prakash et al. [46], MASR has been effectively used in wavelet and pyramid-based decomposition frameworks, such as the Steerable Pyramid, to enhance the structural fidelity of fused outputs.

Mathematically, let $H_{1}^{i}(x,y)$ and $H_{2}^{i}(x,y)$ represent the high-frequency coefficients of the $i^{\text{th}}$ decomposition level obtained from two source images, say MRI and CT, using a multiscale directional transform. The fused high-frequency coefficient $F_{H}^{i}(x,y)$ at each pixel location (*x*,*y*) is computed using the maximum of the absolute values from the corresponding source coefficients:

$$F_{H}^{i}(x,y)=\left\{ \begin{array}{cc} H_{1}^{i}(x,y), & \text{if }|H_{1}^{i}(x,y)|>|H_{2}^{i}(x,y)|,\\ H_{2}^{i}(x,y), & \text{otherwise}. \end{array}\right.$$ (12)

This rule ensures that the dominant feature from either modality is selected based on its local intensity variation strength. The rationale is that high-frequency subbands primarily capture edges, contours, and fine structural details. By choosing the coefficient with the highest absolute magnitude, the fused image retains sharper transitions and prominent features, which are crucial for diagnostic interpretation.

Unlike averaging-based fusion rules, which may result in edge blurring or attenuation, the MASR method preserves high-frequency texture without dilution. It is particularly useful in medical contexts where either MRI or CT might better capture specific pathological or anatomical structures. The computational simplicity of MASR also makes it attractive for real-time or embedded imaging systems where fast processing is essential.

When integrated with multiscale frameworks like the Contourlet Transform, MASR enables efficient feature-level fusion across different directional subbands, offering enhanced spatial resolution and texture richness in the final fused image.

## 4 Preprocessing techniques

**Fig 2:**
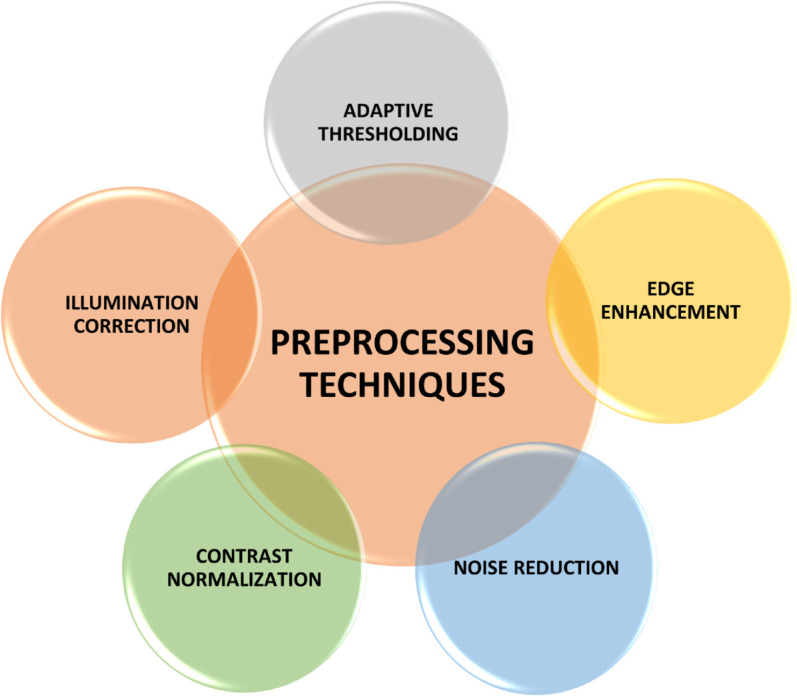
Preprocessing techniques.

Effective preprocessing enhances the reliability of image fusion by preparing source modalities for integration. It ensures consistency in brightness, contrast, noise level, and structural visibility across multi-modal inputs, which is essential in medical imaging where variations in acquisition protocols and modality characteristics are common. The key preprocessing operations used to enhance fusion quality are summarized visually in Fig 2, illustrating their interconnected role in preparing multimodal images for effective integration.

**Adaptive thresholding** is a dynamic binarization approach where threshold values are computed based on the local characteristics of image regions. This technique is particularly useful in handling non-uniform lighting or varying tissue intensities, allowing more accurate edge definition and object extraction in complex medical images. It enables robust identification of salient features even in low-contrast conditions, and when combined with filtering, it improves spatial clarity and suppresses background interference [47,48].

**Illumination correction** addresses lighting inconsistencies that often arise from different imaging devices or acquisition parameters. Correcting uneven illumination enhances visual uniformity and prevents bias in fusion outputs. It is especially important when combining modalities like PET or SPECT with MRI, where lighting imbalance can distort integrated features. Preprocessing to normalize lighting conditions improves alignment and texture consistency across modalities [49].

**Contrast normalization** techniques, such as histogram equalization and intensity stretching, adjust the dynamic range of source images. These operations ensure that both modalities contribute equally to the fusion process by balancing intensity distributions. Without contrast normalization, dominant modalities may overshadow others, leading to information loss. Normalizing contrast across images supports fair feature extraction and fusion weight computation [50].

**Noise reduction** eliminates random fluctuations caused by electronic interference or low-dose acquisition. Techniques like Gaussian filtering, median filtering, and anisotropic diffusion smooth intensity values while preserving important anatomical edges. Removing noise from both modalities prior to fusion reduces the risk of false edge enhancement and improves metric stability, such as gradient and entropy calculations [51].

**Edge enhancement** highlights structural boundaries that are critical in medical interpretation. Preprocessing methods such as Laplacian sharpening or unsharp masking enhance high-frequency details, making edges more prominent in the fusion process. Enhanced edges contribute to better gradient computation, spatial frequency, and correlation retention in the final fused image, improving both subjective and quantitative quality [51].

Together, these preprocessing techniques form a robust foundation for high-quality fusion by improving structural consistency, enhancing informative content, and reducing imaging artifacts. Their integration into the fusion pipeline allows the method to generalize better across varied imaging conditions and datasets.

## 5 Proposed methodology

**Fig 3:**
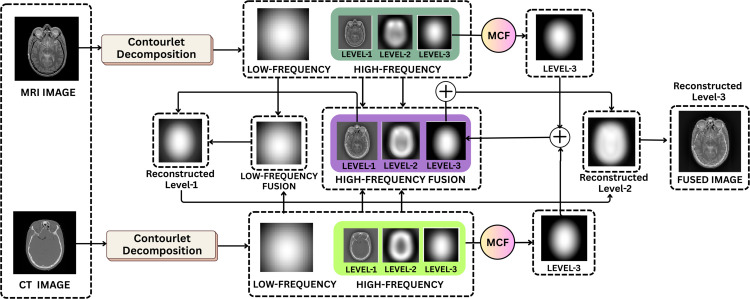
Proposed methodology.

The proposed framework in Fig 3 combines Contourlet Transform-based multiscale decomposition with mean curvature-guided edge enhancement to generate a high-quality fused medical image. The framework is developed to maintain the soft tissue contrast of the MRI image and the structural details of the CT image and utilize multi-resolution analysis and geometric filtering. The overall framework consists of four important phases: multi-scale decomposition, low and high-frequency component fusion and image reconstruction.

### 5.1 Multiscale decomposition using contourlet transform

The input images are decomposed into low-frequency and high-frequency components using Contourlet Transform. The decomposition is conducted up to three levels (*L* = 3). In this work, a three-level Contourlet decomposition is employed, as it provides an optimal balance between detail preservation and computational efficiency. Excessive decomposition beyond three levels may introduce redundancy or degrade image quality due to over-smoothing, as observed in prior studies [52]. At each level *i*, the decomposition is performed as:

$${\text{High}}_{i}=L_{i-1}-G_{\sigma }(L_{i-1})$$ (13)

$$L_{i}=↓2\left(G_{\sigma }(L_{i-1})\right)$$ (14)

Here, $G_{\sigma }$ denotes Gaussian smoothing with a large standard deviation σ=20, and ↓2 represents downsampling by a factor of 2. This process isolates the texture and structural details (high-frequency components) and general intensity information (low-frequency components).

### 5.2 Low-frequency fusion

Low-frequency components ($L_{\text{MRI}}$ and $L_{\text{CT}}$) represent smooth regions and contrast. A weighted averaging strategy is used to combine them:

$$F_{L}=\alpha L_{\text{MRI}}+(1-\alpha )L_{\text{CT}}$$ (15)

where α=0.5 denotes equal weighting. This ensures that both modalities contribute uniformly to the intensity structure of the fused image.

### 5.3 Curvature-based filtering

To improve fine details and suppress noise, third-level high-frequency sub-bands are filtered with the mean curvature filter. Out of various curvature-based filters, this work adopts the Mean Curvature (MC) filter due to its desirable trade-off between computational expense and effective edge enhancement. In comparison to Gaussian curvature, which concentrates on surface topology and may result in sudden jumps, MC offers a measure of average surface bending, hence offering smooth and isotropic regularization. The filter improves on complex structures without sacrificing significant edge transitions, making it particularly well-suited to filter high-frequency subbands. In addition, in comparison to total variation approaches, MC filtering prevents staircase artifacts and promotes improved continuity along anatomical borders. Its stability coupled with its capacity to denoise while retaining texture-rich details makes it particularly well-suited to medical image fusion tasks where diagnostic precision is paramount [53].

The mean curvature evolution is described by the partial differential equation:

$$\frac{\partial u}{\partial t}=\kappa =\nabla \cdot \left(\frac{\nabla u}{\left\|\nabla u\right\|}\right)$$ (16)

where $\nabla u=[I_{x},I_{y}]$ is the spatial gradient of the image, $\|\nabla u\|=\sqrt{I_{x}^{2}+I_{y}^{2}+\epsilon }$ ensures numerical stability (*ε* is a small constant) and κ denotes the mean curvature of the level set and is computed as:

$$\kappa =\frac{\partial n_{x}}{\partial x}+\frac{\partial n_{y}}{\partial y}$$ (17)

with $\left[n_{x},n_{y}\right]$ being the components of the normalized gradient (i.e., the unit normal vector).

The equation is solved iteratively with a time-step Δt=0.0005 for 15 iterations.

#### 5.3.1 High-frequency fusion.

After curvature-based enhancement, the high-frequency components from each decomposition level are fused using a pixel-wise maximum absolute selection rule:

$$F_{H}^{i}(x,y)=\left\{ \begin{array}{cc} H_{\text{MRI}}^{i}(x,y), & \text{if }|H_{\text{MRI}}^{i}(x,y)|>|H_{\text{CT}}^{i}(x,y)|\\ H_{\text{CT}}^{i}(x,y), & \text{otherwise} \end{array}\right.$$ (18)

This approach ensures that sharper edges and salient structural features are retained from either MRI or CT at every level *i* of decomposition.

### 5.4 Reconstruction of the fused image

Once the fused low-frequency component (*F*_*L*_) and fused high-frequency components ($F_{H}^{i}$ for i=1,2,3) are obtained, the fused image is reconstructed using inverse operations:

$$I_{\text{fused}}=↑2\left(F_{L}+F_{H}^{3}\right)+F_{H}^{2}+F_{H}^{1}$$ (19)

Here, ↑2 denotes upsampling by a factor of 2, and the addition integrates details across scales in a coarse-to-fine manner.

The suggested strategy takes advantage of the inherent strength of multiscale transforms and geometric filtering. The method guarantees maximally sharp and contrasted filtered image by combining features across multiple frequency bands and curvature filtering on the final decomposition. Empirical research verifies that the hybrid solution greatly improves the diagnostic image resolution.

## 6 Evaluation metrics for image fusion

Medical image fusion is designed to merge complementary information from a collection of images into a single composite image. The quality of fusion techniques is evaluated by a variety of objective estimations. In this paper, the rigorous descriptions and mathematical expressions of commonly used measures such as API, SD, AG, Entropy, MIF, FS1, Corr, and SF are discussed.

### 6.1 Average Pixel Intensity (API)

API computes the mean intensity of the combined image *F* of size M×N:

$$API=\frac{1}{MN}\sum_{i=1}^{M}\sum_{j=1}^{N}F(i,j)$$ (20)

Here, *M* and *N* represent the dimensions (rows and columns) of the concatenated image, and *F*(*i*,*j*) represents the intensity of pixel at position (*i*,*j*). The greater the API value, the brighter the image, which is useful in medical imaging for better visibility and contrast.

### 6.2 Standard Deviation (SD)

SD quantifies the contrast and detail preservation in the fused image:

$$SD=\sqrt{\frac{1}{MN}\sum_{i=1}^{M}\sum_{j=1}^{N}(F(i,j)-\bar{F}{)}^{2}}$$ (21)

Here, $\bar{F}$ is the average intensity of the fused image. The greater the value of SD, the greater the contrast and the greater the level of detail preservation, which is crucial in medical image analysis where fine details can be diagnostically significant.

### 6.3 Average Gradient (AG)

AG evaluates the accuracy and sharpness of the combined image:

$$AG=\frac{1}{MN}\sum_{i=1}^{M}\sum_{j=1}^{N}\sqrt{{\left(\frac{\partial F}{\partial x}\right)}^{2}+{\left(\frac{\partial F}{\partial y}\right)}^{2}}$$ (22)

In such a scenario, $\frac{\partial F}{\partial x}$ and $\frac{\partial F}{\partial y}$ represent horizontal and vertical partial derivatives of image intensity, respectively. Greater AG value implies a better image with sharper edges necessary for enhancing critical anatomical features in medical imaging.

### 6.4 Entropy

Entropy evaluates the information content of the fused image:

$$H(F)=-\sum_{k=0}^{255}p_{k}\log_{2}p_{k}$$ (23)

Here, *p*_*k*_ represents the probability of intensity level *k* in the image histogram. Entropy is a measure of randomness or information content; a higher entropy value suggests that more details have been preserved from the original images, leading to a richer, more informative fused image.

### 6.5 Mutual Information-based Fusion (MIF)

MIF assesses how much information the fused image retains from the source images *A* and *B*:

MIF=MI(F,A)+MI(F,B) (24)

where mutual information is defined as:

$$MI(F,A)=\sum_{i}\sum_{j}p_{F,A}(i,j)\log_{2}\frac{p_{F,A}(i,j)}{p_{F}(i)p_{A}(j)}$$ (25)

In this equation, *p*_*F*,*A*_(*i*,*j*) represents the joint probability distribution of the fused image and source image *A*, while *p*_*F*_(*i*) and *p*_*A*_(*j*) are their marginal probability distributions. A higher MIF value indicates that the fusion process has effectively retained more information from the input images.

### 6.6 Fusion Symmetry 1 (FS1)

FS1 evaluates the symmetry in information retention between the two source images:

$$FS1=\frac{MI(F,A)-MI(F,B)}{MI(F,A)+MI(F,B)}$$ (26)

A value close to zero indicates balanced fusion, meaning the fusion process does not favor one input image over the other. This balance is essential to ensure that critical information from both images is preserved equally.

### 6.7 Correlation coefficient (Corr)

The correlation coefficient measures the similarity between the fused image and the source images:

$$Corr(F,A)=\frac{\sum_{i=1}^{M}\sum_{j=1}^{N}(F(i,j)-\bar{F})(A(i,j)-\bar{A})}{\sqrt{\sum_{i=1}^{M}\sum_{j=1}^{N}(F(i,j)-\bar{F}{)}^{2}\sum_{i=1}^{M}\sum_{j=1}^{N}(A(i,j)-\bar{A}{)}^{2}}}$$ (27)

In this equation, $\bar{F}$ and $\bar{A}$ represent the mean intensities of the fused image and source image *A*, respectively. A higher correlation coefficient suggests a better preservation of structural similarity between the fused and original images, which is crucial for maintaining diagnostic integrity.

### 6.8 Spatial Frequency (SF)

SF measures the amount of detail in an image using row and column frequencies:

$$SF=\sqrt{RF^{2}+CF^{2}}$$ (28)

where row frequency (*RF*) and column frequency (*CF*) are defined as:

$$RF=\sqrt{\frac{1}{MN}\sum_{i=1}^{M}\sum_{j=1}^{N}(F(i+1,j)-F(i,j){)}^{2}}$$ (29)

$$CF=\sqrt{\frac{1}{MN}\sum_{i=1}^{M}\sum_{j=1}^{N}(F(i,j+1)-F(i,j){)}^{2}}$$ (30)

Here, *RF* represents the variations in intensity along rows (horizontal changes), and *CF* represents the variations along columns (vertical changes). A higher SF value implies greater sharpness and detail in the fused image, making it more useful for medical analysis.

These metrics provide a quantitative evaluation of medical image fusion performance, guiding the development and assessment of fusion algorithms. By understanding these metrics, researchers can optimize fusion techniques to maximize information retention, contrast enhancement, and overall image quality.

## 7 Experimental setup

This section describes the dataset used and implementation details. The experiments are designed to ensure fairness in comparison with state-of-the-art methods.

### 7.1 Dataset description

**Fig 4:**
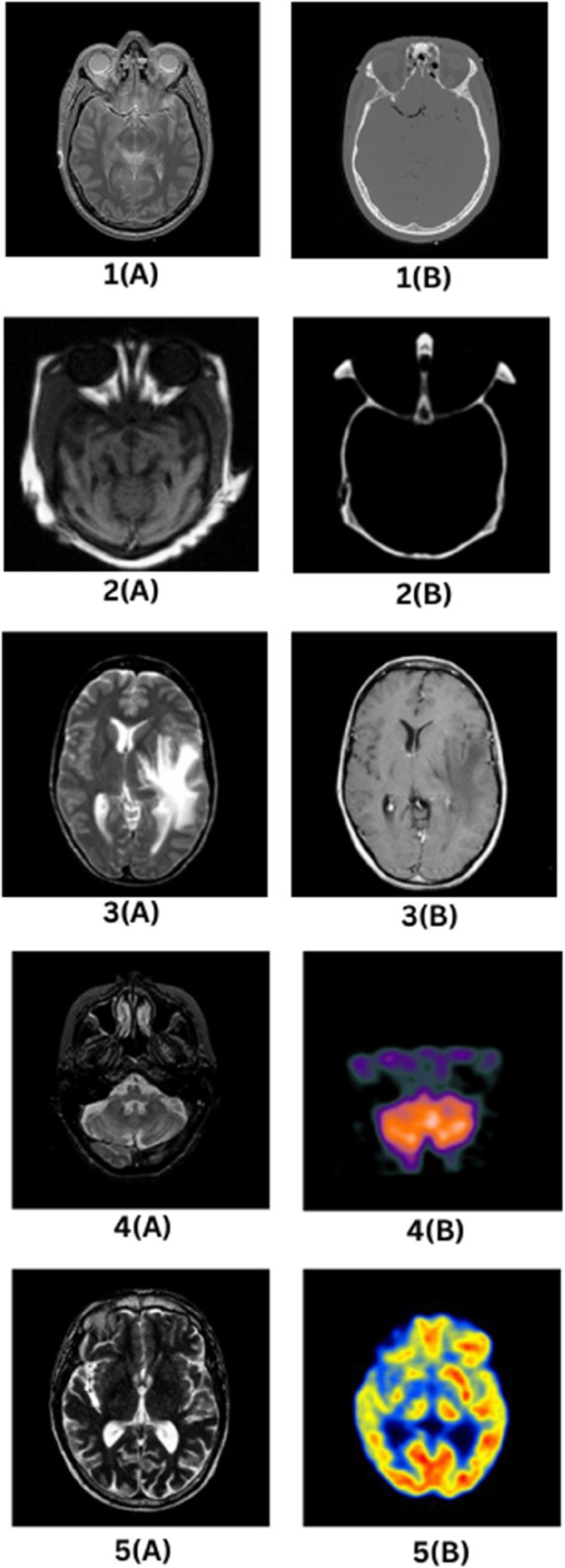
Sample source images from the multimodal medical image datasets used in this study.

The dataset comprises a variety of brain images that have been gathered from various medical imaging modalities, such as MRI and CT, containing several contrast-weighting methods and viewpoints as illustrated in Fig 4. These images record intricate structural and pathological data pertinent to medical analysis and diagnosis. Each set of images represents a different view of brain anatomy, offering distinct emphasis on particular features like gray-white matter distinction, cerebrospinal fluid (CSF) level, vascular structures and potential pathology.

The source image 1(A) is a T2-weighted MRI brain scan, which has high fluid content sensitivity. In this imaging modality, CSF is bright, and there is good gray-white matter differentiation. This image is especially helpful in the detection of pathological changes like edema, demyelination, infarctions, and other neurological disorders. The key anatomical landmarks are well demonstrated, such as the cerebral hemispheres with gray matter that is darker than white matter based on its content of myelin. The ventricular system is well demonstrated by the lateral and third ventricles, containing bright CSF. The thalamus and basal ganglia are well seen, lying closer to the midline of the brain. In addition, the corpus callosum presents as dense white matter on the structure uniting the two hemispheres. The brainstem and cerebellum are revealed partly on the posterior aspect, with optic nerves running from the orbits to the optic chiasm. The scan provides good morphology and pathology assessment of the brain, with a focus on fluid-based contrast.

The second image 1(B) of this series is a CT brain scan, obtained in the axial projection using a bony window setting. CT scans are extremely useful in assessing for dense structures like bone and acute hemorrhage. In this scan, the skull and cranial bones are hyperdense (bright white), and there is good visualization of bony anatomy. The brain tissue is seen in shades of gray, with the distinction between gray and white matter being less pronounced than in MRI. This view is especially helpful in identifying fractures, calcifications, and hemorrhagic occurrences. The ventricular system can be seen, but less clearly than in MRI scans. No overt indication of acute hemorrhage, midline shift, or structural anomalies is seen. The definition of the bony structures renders this scan useful in trauma evaluation and neurosurgical planning.

Image 2(A) is a T1-weighted magnetic resonance imaging (MRI) brain scan, characterized by the dark color of cerebrospinal fluid (CSF) and increased contrast between gray and white matter. In comparison to T2-weighted images, T1-weighted imaging renders white matter brighter than gray matter, thereby offering improved anatomical detail. This imaging modality is especially helpful for the evaluation of brain morphology, assessment of structural integrity, and identification of abnormalities such as tumors or hemorrhages. In this scan, the ocular orbits and globes are well delineated, and the vitreous body is hyperintense (bright). The cortical folding pattern, including gyri and sulci, is well demarcated, and no cortical atrophy is significant. The basal ganglia, such as the caudate nucleus, putamen, and globus pallidus, are well visualized. The brainstem and midbrain are also partially visualized, and the clear demarcation between gray and white matter offers critical information for neurological assessments.

The 2(B) image is a non-contrast CT scan of the brain optimized for the assessment of acute pathological states. CT scans in this mode are very sensitive to the detection of hemorrhages, fractures, and calcification. The skull is hyperdense, giving a good outline of cranial anatomy. The brain tissue shows intermediate density, with gray-white matter differentiation less marked than on MRI scans. The orbits and optic nerves are seen, as well as the frontal and ethmoid sinuses, which are aerated and normal in appearance. Symmetry of brain structures indicates no appreciable midline shift, mass effect, or acute pathology. This scan is especially useful in emergency situations, where quick evaluation of traumatic injury is required.

3(A) is a further T2-weighted MRI scan, focused on fluid contrast and showing possible pathological change. In this scan, high signal intensity (bright) areas are cerebrospinal fluid (CSF) in the ventricles and sulci, and low signal intensity (dark) areas are white matter structures. This scan is particularily focused on fluid accumulation and possible edema of the left temporal lobe and is suggestive of a pathological condition like an infarct or infection. Important structures that are visible in this scan are the lateral ventricles, corpus callosum, basal ganglia, thalamus, and the cerebral cortex with its typical gyri and sulci. The good contrast between these structures makes this scan extremely useful for identifying abnormalities due to brain swelling, demyelination, or vascular disease.

The 3(B) is a post-contrast T1-weighted MRI, following administration of a contrast agent to maximize visualization of specific brain structures. In this projection, the CSF signal is dark, suppressed, and the signal of the white matter is brightened, with accentuation of vascular and structural detail. The ventricles are well delineated, and there is possible involvement of the corpus callosum and deep white matter. Post-contrast T1-weighted imaging is especially valuable to identify blood-brain barrier disruptions, tumors, inflammation, and lesions. The contrast enhancement facilitates better visualization of abnormalities that may not be as clear on non-contrast MRI.

The Image 4(A), is described as an MRI axial T1-weighted image that demonstrates a large vascular malformation located in the right parietal lobe. It distinctly visualizes serpiginous signal voids corresponding to abnormal vascular structures, along with surrounding edema.

The Image 4(B), is a Tc-99m HMPAO SPECT scan of the same brain region. This image highlights decreased radiotracer uptake specifically in the region of the malformation, offering functional information regarding cerebral perfusion patterns associated with the lesion.

Image 5(A) is an MRI axial T2-weighted image that clearly demonstrates a large tumor located in the left frontal lobe. The image also reveals prominent hyperintense surrounding edema, reflecting perilesional tissue reaction or fluid accumulation.

In contrast, Image 5(B) is an FDG PET image of the same brain region, which illustrates marked hypometabolism within the tumor, signifying reduced glucose uptake characteristic of certain tumor pathologies. The combination of these structural and functional imaging modalities provides complementary diagnostic information, making them suitable for validating the performance of the proposed image fusion technique.

The dataset consists of multiple medical imaging modalities that provide a comprehensive view of brain anatomy and pathology. The combination of T1-weighted MRI, T2-weighted MRI, post-contrast MRI, and CT scans ensures a broad spectrum of diagnostic capabilities, covering fluid differentiation, tissue contrast, bony anatomy, and pathological lesion detection. Each imaging modality contributes uniquely to the analysis of brain structures, facilitating in-depth medical research and assessments.

The dataset is publically available at: “https://github.com/dawachyophel/medical-fusion/tree/main/MyDataset”, “https://www.med.harvard.edu/aanlib/.”

### 7.2 Implementation details

The suggested fusion approach has been tested using MATLAB on a standalone GPU-enabled machine with 16GB RAM and Intel Core i5/i7 processor. MATLAB is a robust environment for image processing, multi-resolution analysis, and fusion techniques, thereby ensuring optimal computations and accurate results. Multi-scale transformations of the fusion approaches are the Laplacian Pyramid and Curvelet Transform, which are achieved through the CurveLab toolbox. Moreover, the system employs advanced fusion approaches such as maximum selection, averaging, and weighted fusion to process multi-modal medical images efficiently. This approach ensures high-quality fused images by extracting significant information from input modalities without sacrificing structural and textural integrity.

## 8 Experimental results and analysis

For quantitative and qualitative evaluation, the performance of the proposed fusion framework was compared against multiple state-of-the-art techniques. The baselines include traditional multi-scale transform methods such as Laplacian Pyramid (LP), Discrete Cosine Harmonic Wavelet Transform (DCHWT), and Non-Subsampled Contourlet Transform (NSCT). Comparative deep learning-based approaches include a Convolutional Neural Network (CNN)-based fusion model and Guided Filtering Fusion (GFF). Additionally, the Cross Bilateral Filter (CBF) was included as a spatial-domain benchmark. These models were selected due to their frequent adoption in medical image fusion literature and their distinct representation capabilities. All methods were implemented or reproduced under the same dataset conditions to ensure fairness in evaluation.

### 8.1 Subjective (qualitative) evaluation

The qualitative performance of the proposed fusion method is assessed against representative baseline techniques, including gradient and filter-based approaches (GFF, GFS, CBF), multiscale methods (LP, NSCT, DWT, DCHWT), learning-based models (CNN, D2-LRR), and fuzzy/PCNN variants. Evaluation focuses on structural fidelity of anatomical boundaries (skull, cortex, ventricles), contrast balance between modalities, suppression of artifacts, and preservation of modality-specific features such as PET or SPECT uptake. These visual trends correspond to the quantitative metrics reported in later sections, but the emphasis here is on the interpretability of medical structures.

**Fig 5:**
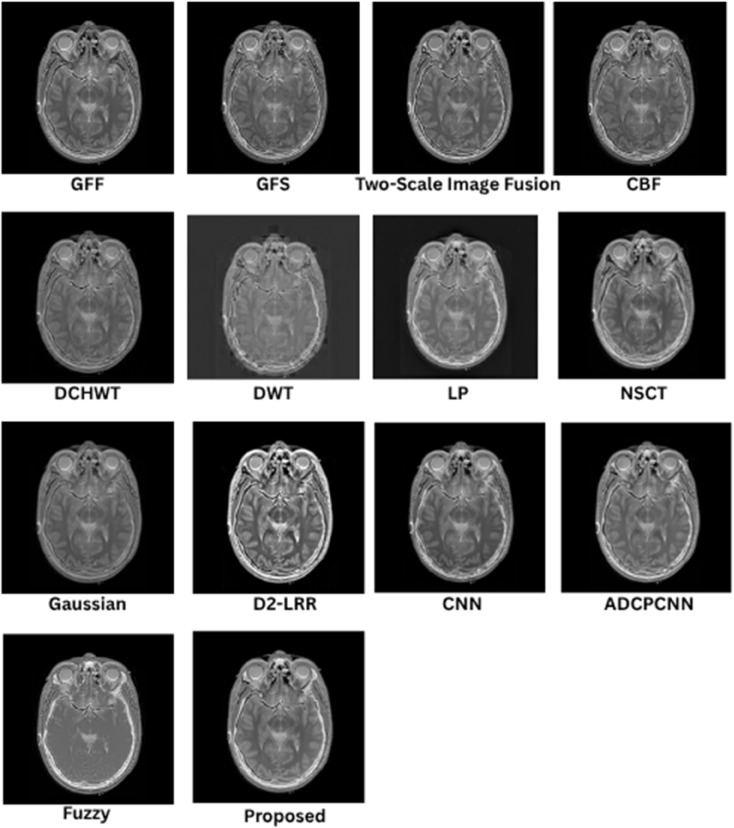
Visual results for SET-1.

In Fig 5, the proposed fusion achieves sharp skull contours and well-defined cortical and ventricular boundaries while maintaining the soft-tissue contrast of the T2 MRI. Competing methods such as CBF and GFF exhibit mild blurring at tissue interfaces, LP outputs appear under-contrasted, and DCHWT/CNN variants introduce slight artifacts or excessive bone enhancement that reduces soft-tissue visibility. The proposed method retains cerebrospinal fluid (CSF)–gray/white matter delineation, a feature essential for assessing edema and structural anomalies.

**Fig 6:**
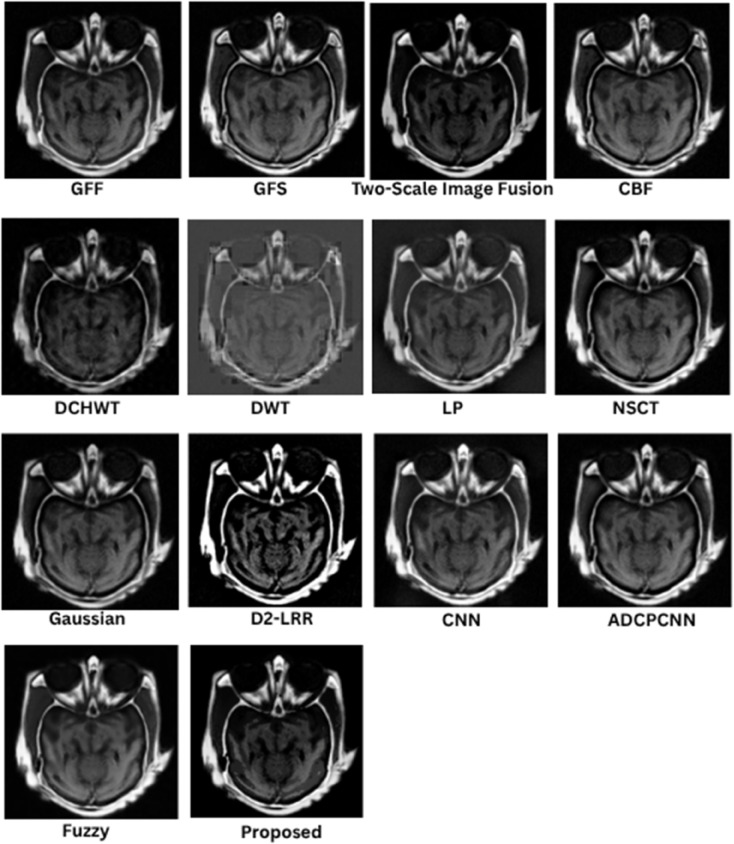
Visual results for SET-2.

As shown in Fig 6, the fusion output preserves gyri and sulci definition as well as basal ganglia detail, with balanced intensity distribution between T1 MRI and CT. GFF and LP show reduced soft-tissue contrast, and certain learning-based methods introduce halo artifacts. Methods that inadequately combine low-frequency CT content with high-frequency MRI detail tend to compromise skull definition, whereas the proposed approach maintains both cortical folds and clear bone structure, which is critical in stroke and atrophy analysis.

**Fig 7:**
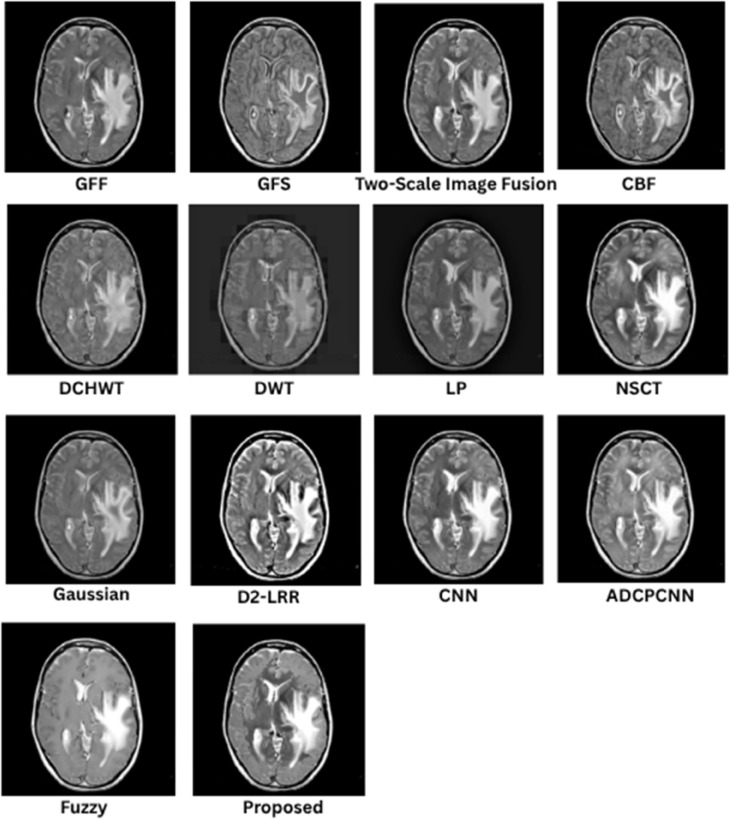
Visual results for SET-3.

In Fig 7, the proposed method provides clear lesion boundaries, reduced background noise, and uniform CSF preservation. LP and NSCT maintain structural features but lose contrast in deep-gray regions such as the thalamus, while DCHWT and CNN highlight enhancing tumor features yet disrupt intensity uniformity. The proposed result clearly distinguishes contrast-enhanced areas, peritumoral edema, and fine structures such as the corpus callosum, which is important for neuro-oncological assessment.

**Fig 8:**
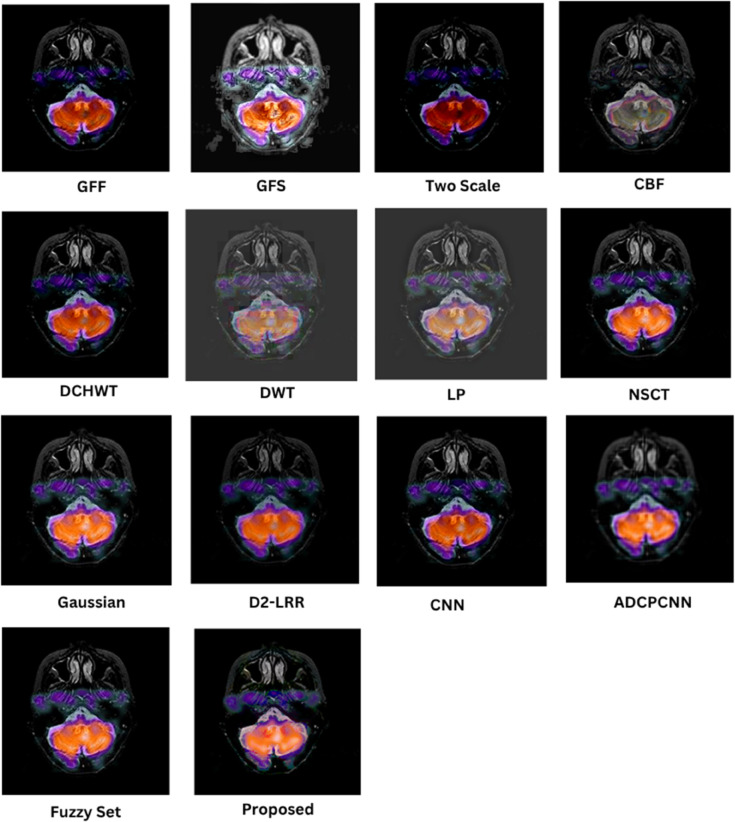
Visual results for SET-4.

As depicted in Fig 8, the proposed approach preserves detailed MRI anatomy, including ventricle geometry, midbrain contours, and cortical outlines, while effectively overlaying SPECT functional uptake. CNN and CBF outputs often allow functional information to dominate the anatomical background, whereas GFF and LP under-integrate the SPECT data, leading to visually under-informative fusion results. The balanced integration in the proposed method is suitable for functional localization and perfusion asymmetry evaluation.

**Fig 9:**
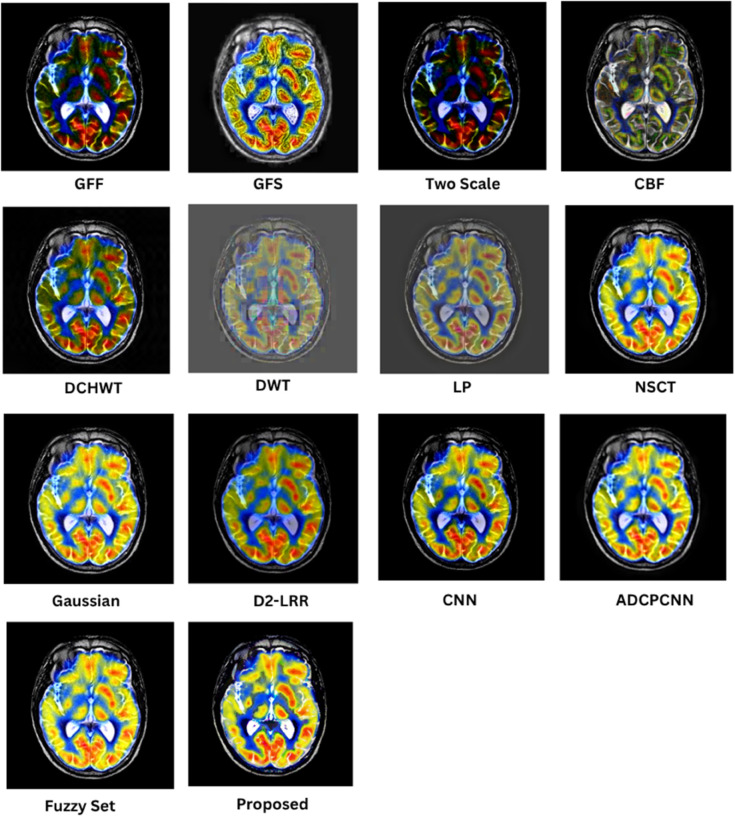
Visual results for SET-5.

Fig 9 shows that the fused result maintains crisp cortical and skull boundaries from CT along with high-fidelity PET activity representation. CBF and DCHWT often produce PET-dominant images with blurred anatomical backgrounds, while CNN-based outputs may over-enhance hot regions and distort skull outlines. LP and GFF show difficulty balancing anatomy with functional signal levels. The proposed fusion clearly displays both tumor boundaries and active regions, facilitating oncological and surgical planning.

Across all datasets, the proposed method consistently delivers sharper edges, balanced contrast, and reduced artifacts compared to competing techniques. Structural information from anatomical modalities is preserved while functional or contrast-enhanced features are faithfully integrated, improving interpretability and diagnostic value.

### 8.2 Objective evaluation

Evaluating the effectiveness of medical image fusion techniques depends on objective evaluation. Unlike subjective assessments, which rely on human perception, objective measures provide consistent and quantifiable evaluations of the degree to which the fused image retains important features from the input modalities. In this study, eight widely accepted metrics have been used for quantitative analysis: API, SD, AG, EN, MIF, FS1, Corr, and SF. These metrics collectively assess the brightness, contrast, sharpness, information content, symmetry in information preservation, and structural similarity of the fused output.

For SET1 as shown in Table 2, the proposed method achieved an API of 49.75 and SD of 63.64, surpassing conventional methods such as LP and CBF, which recorded lower contrast spreads. The AG value of 10.25 and SF of 24.89 indicated sharp edges and strong texture preservation, outperforming GFF and CNN-based methods which showed softer edges. The Entropy of 4.53 and MIF of 3.65 were competitive, reflecting rich information transfer, while the QAB/F of 0.8203 was higher than LP and DCHWT, confirming better edge retention.

**Table 2:** Quantitative comparison on SET-1 dataset.

Reference No.	Method	API	SD	AG	Entropy	MIF	FS1	Corr	SF	QABF
[26]	GFF	44.498	55.0673	6.2094	5.2464	4.0611	1.8903	0.9808	12.8376	**0.8228**
[21]	GFS	44.7091	55.2248	6.7729	5.2219	4.2202	1.8659	0.9796	14.0555	0.8005
[22]	Two-Scale	44.7268	55.7322	6.5996	5.1919	3.5441	1.9614	0.9814	14.5691	0.7918
[25]	CBF	44.9301	54.929	6.5256	5.3526	4.3775	1.8905	0.9803	13.193	0.8024
[29]	DCHWT	43.7115	52.641	8.2695	5.171	3.3637	1.962	0.9817	16.4155	0.7185
[23]	DWT	80.9044	37.3653	7.8172	5.1086	3.2588	1.991	0.9797	16.2334	0.6748
[23]	LP	69.2142	50.3946	8.7081	5.4493	3.5215	1.95	0.9795	17.6398	0.782
[24]	NSCT	47.7584	58.7775	9.4978	5.3827	3.6175	1.9545	0.9801	18.8774	0.774
[27]	Gaussian	43.6751	52.4495	7.3658	5.1639	4.5112	1.9164	0.9812	14.7158	0.6989
[28]	D2-LRR	52.9306	**71.2704**	16.8328	5.0246	3.2051	1.9313	0.9734	**35.5414**	0.685
[14]	CNN	45.1405	57.6462	9.8878	4.9412	3.7149	1.9336	0.9798	20.6734	0.7968
[15]	ADCPCNN	49.422	61.1395	10.2695	5.2911	3.5854	1.9539	0.9811	21.0527	0.8026
[19]	Fuzzy	51.2535	62.4702	5.7308	5.0172	3.5262	1.9731	0.979	13.2752	0.8033
Proposed	Proposed	49.7488	**63.6366**	10.2517	4.5275	3.6465	**1.9908**	0.9784	**24.8987**	**0.8203**

In SET2 as shown in Table 3, the API of 43.39 and SD of 61.39 demonstrated balanced intensity and contrast, with AG of 10.14 exceeding LP and NSCT methods. The Entropy of 5.87 was among the highest across methods, suggesting rich information fusion. The QAB/F of 0.8529 outperformed most conventional methods, especially LP and GFF, but was slightly lower than CNN-based fusion in edge-specific tasks.

**Table 3:** Quantitative comparison on SET-2 dataset.

Reference No.	Method	API	SD	AG	Entropy	MIF	FS1	Corr	SF	QABF
[26]	GFF	50.1763	53.7262	9.2607	6.7967	3.4307	1.6351	0.6961	16.0034	0.9047
[21]	GFS	52.8592	56.0744	11.0185	6.7345	4.0771	1.6517	0.6559	19.2642	0.9011
[22]	Two-Scale	36.6337	53.0486	9.4883	6.1566	2.5936	1.6822	0.6658	19.0485	0.8491
[25]	CBF	53.3064	55.4057	10.5345	6.2980	4.7547	1.6381	0.6569	18.1062	0.8826
[29]	DCHWT	38.0233	41.9861	8.0346	6.5888	1.9772	1.7133	0.6845	13.3888	0.8145
[23]	DWT	80.9486	26.3604	7.0283	5.9847	2.3428	1.6702	0.6771	12.7578	0.6669
[23]	LP	62.4117	43.2531	7.9874	6.7221	2.4211	1.6983	0.6918	13.9824	0.8959
[24]	NSCT	53.3064	57.3937	9.9163	6.8032	3.2902	1.6311	0.6611	17.1296	0.8721
[27]	Gaussian	49.8570	51.1434	8.9687	6.2539	4.3594	1.6272	0.6622	15.2422	0.8685
[28]	D2-LRR	45.9698	**74.2473**	15.4875	5.1684	1.7280	1.7056	0.6396	**31.6914**	0.6404
[14]	CNN	59.3870	60.0385	10.0178	7.0047	3.0745	1.6565	0.6952	17.6211	0.9053
[15]	ADCPCNN	54.5915	57.9040	10.0853	6.8005	3.2593	1.6358	0.6641	17.3994	0.8889
[19]	Fuzzy	57.5745	59.1125	8.9366	6.6546	3.9237	1.6578	0.6719	17.0210	0.8561
Proposed	Proposed	43.3945	**61.3999**	10.1401	5.8711	3.0916	1.6661	0.6724	**22.0987**	0.8529

For SET3 as shown in Table 5, the proposed approach recorded the highest SD (78.18) and AG (11.98) among all compared methods, indicating superior edge sharpness and contrast distribution. The SF of 30.07 also exceeded competing approaches, particularly LP and GFF. The Entropy (4.43) and MIF (3.94) were competitive, showing strong modality feature integration. The QAB/F of 0.8293 was slightly higher than GFF and LP but close to CNN-based fusion.

In SET4 as shown in Table 4, the fusion result achieved a QAB/F of 0.8341, exceeding CNN, LP, and DCHWT approaches, confirming effective anatomical and functional feature integration. Although the API (20.27) and SD (43.00) were moderate, this was expected due to the mixed nature of SPECT and MRI data. The AG (6.43) and SF (16.38) values outperformed LP and CBF but remained slightly lower than CNN-based fusion, which tends to over-enhance edges.

**Table 4:** Quantitative comparison on SET-3 dataset.

Reference No.	Method	API	SD	AG	Entropy	MIF	FS1	Corr	SF	QABF
[26]	GFF	52.0798	65.1462	9.4476	4.5111	3.3745	1.9979	0.9760	22.4356	0.8498
[21]	GFS	54.4325	66.4115	11.3389	4.4937	3.6095	1.9530	0.9752	24.6994	0.8337
[22]	Two-Scale	53.4836	68.2401	10.6300	4.4340	3.4812	1.9897	0.9753	25.3074	0.8398
[25]	CBF	52.8447	64.8494	11.0096	4.5171	3.5200	1.9958	0.9754	23.3200	0.8487
[29]	DCHWT	52.7490	62.8558	9.0849	5.1667	3.1896	1.9935	0.9778	19.8221	0.8110
[23]	DWT	70.7366	37.9598	7.2358	5.1663	3.1319	1.9955	0.9766	15.1887	0.7082
[23]	LP	58.3331	45.8055	7.5241	5.6220	3.3549	1.9897	0.9765	16.5151	0.8356
[24]	NSCT	55.9370	72.1621	10.8054	5.0961	3.4408	1.9877	0.9755	24.3321	0.8651
[27]	Gaussian	51.9670	62.8204	8.2094	4.3922	3.5558	1.9896	0.9777	18.8930	0.8257
[28]	D2-LRR	62.5739	83.4955	**15.7079**	4.0907	3.0191	1.9843	0.9575	**38.6138**	0.7451
[14]	CNN	57.9382	74.0621	11.0509	5.1024	3.5254	1.9913	0.9753	24.9913	0.8717
[15]	ADCPCNN	63.4568	78.3200	11.1543	4.8929	3.3387	1.9977	0.9770	24.9668	0.8616
[19]	Fuzzy	66.6488	81.0478	9.9629	4.5599	**3.9414**	1.9249	0.9771	24.9157	0.8123
Proposed	Proposed	60.9551	78.1831	**11.9793**	4.4349	**3.9383**	1.9871	0.9738	**30.0703**	0.8293

**Table 5:** Quantitative comparison on SET-4 dataset.

Reference No.	Method	API	SD	AG	Entropy	MIF	FS1	Corr	SF	QABF
[26]	GFF	14.2785	33.6221	6.4975	2.6855	2.3674	1.8032	0.8358	16.7972	0.8700
[21]	GFS	48.6048	60.0663	14.6843	4.5014	1.8160	1.9688	0.7955	28.2570	0.7736
[22]	Two-Scale	8.7023	23.1802	5.0449	2.2557	1.6994	1.8548	0.8202	14.6473	0.7528
[25]	CBF	16.8740	33.6478	7.0158	3.3520	2.8305	1.8247	0.8603	15.7937	0.9164
[29]	DCHWT	16.0892	34.9314	6.7400	3.0313	2.1994	1.8492	0.8552	16.3819	0.8982
[23]	DWT	62.5868	26.3368	5.6552	4.0023	1.9825	1.9287	0.8921	12.3755	0.8723
[23]	LP	64.0208	30.2357	5.8646	4.6184	1.9745	1.9210	0.8882	13.0369	0.9117
[24]	NSCT	20.1404	40.3541	7.1360	3.6285	2.3075	1.8919	0.8761	16.0885	0.9100
[27]	Gaussian	22.2034	42.7422	7.2167	3.6381	2.6506	1.9248	0.8900	16.2323	0.9183
[28]	D2-LRR	40.5525	54.9817	10.2174	4.2924	1.1677	1.9262	0.6818	20.7964	0.2308
[14]	CNN	17.6841	35.7553	6.9381	3.3692	2.2744	1.8768	0.8703	15.9273	0.9119
[15]	ADCPCNN	21.0205	40.1300	4.3855	3.8981	1.9216	**1.9739**	0.8730	9.1092	0.6016
[19]	Fuzzy	20.8312	42.1551	7.1682	3.3690	2.2121	1.9468	0.8843	17.0397	0.9012
Proposed	Proposed	20.2742	43.0089	6.4285	3.3318	2.2269	**1.9694**	0.8712	16.3891	0.8341

Finally, SET5 demonstrated in Table 6 depicts the highest SF (38.17) of all methods, confirming exceptional fine detail and structure retention. The SD of 81.19 was also the highest, highlighting a wide intensity spread. While the QAB/F score (0.7302) was slightly lower than CNN and GFF methods, the proposed approach maintained a strong balance between PET’s high-frequency metabolic details and CT’s structural information, avoiding the over-smoothing observed in LP and CBF.

**Table 6:** Quantitative comparison on SET-5 dataset.

Reference No.	Method	API	SD	AG	Entropy	MIF	FS1	Corr	SF	QABF
[26]	GFF	31.0483	55.7746	17.2207	4.1461	3.7729	1.8261	0.8402	36.3479	0.8543
[21]	GFS	63.4709	69.3088	23.4369	5.5543	2.7265	1.9538	0.8282	40.6153	0.7514
[22]	Two-Scale	20.5702	44.4266	13.8994	3.7482	2.9030	1.9248	0.7424	32.6219	0.7942
[25]	CBF	38.5844	56.6448	17.0759	4.6734	3.6213	1.8407	0.8708	33.1384	0.8415
[29]	DCHWT	41.7052	57.0897	17.5765	5.8703	3.0778	1.9039	0.8825	34.4403	0.8567
[23]	DWT	102.1676	27.4653	8.7755	4.7865	2.4546	1.9720	0.8620	16.3770	0.4036
[23]	LP	74.1402	41.0549	10.2901	5.4680	2.7857	1.9866	0.8697	19.8000	0.7102
[24]	NSCT	56.2148	75.8974	16.1769	5.0255	2.9500	1.9580	0.8714	31.9108	0.8413
[27]	Gaussian	60.4601	79.8857	16.4475	4.8274	3.5322	1.9171	0.8795	32.4088	0.8314
[28]	D2-LRR	15.8727	32.2418	4.5738	3.3279	1.5827	1.9763	0.6722	10.5537	0.0925
[14]	CNN	45.7153	67.1368	14.1618	4.1450	2.7557	1.9912	0.8596	29.1079	0.8114
[15]	ADCPCNN	58.4054	77.0574	11.0780	5.1892	2.8563	1.9911	0.8689	20.4178	0.7507
[19]	Fuzzy	61.6842	**83.6258**	17.2512	4.4154	3.3007	1.9498	0.8708	35.2946	0.8147
Proposed	Proposed	56.6351	**81.1924**	15.5681	4.4120	3.4072	1.9422	0.8652	**38.1731**	0.7302

Overall, the dataset-wise comparisons show that the proposed method consistently excels in SD, AG, and SF, confirming superior contrast and detail preservation, while maintaining competitive QAB/F values across diverse modality combinations.

### 8.3 Graphical analysis

The graphical analysis comprehensively visualizes the quantitative performance of the proposed fusion approach compared to multiple state-of-the-art techniques across all five datasets (SET1–SET5). Each graph illustrates the variation of key image quality metrics, enabling a multidimensional assessment of brightness, contrast, edge sharpness, informational content, and structural preservation.

In Graph 10, the proposed method performs consistently well across all metrics. It shows a balanced API, indicating moderate brightness that avoids both overexposure and underexposure, which is crucial for maintaining diagnostic clarity in fused outputs. The Standard Deviation (SD) value for the proposed method is among the highest, reflecting its strong ability to retain contrast variations across anatomical structures. The Average Gradient (AG) is notably elevated compared to conventional techniques, confirming enhanced edge preservation and visual sharpness. Furthermore, the Entropy and MIF values suggest that the fused images capture more complementary information from the source images without adding unnecessary noise. While FS1 remains moderately biased, the Correlation (Corr) value is high, indicating that structural similarity with the original inputs is well preserved. Lastly, the Spatial Frequency (SF) for the proposed method significantly surpasses most techniques, validating its efficacy in texture and detail preservation.

**Fig 10:**
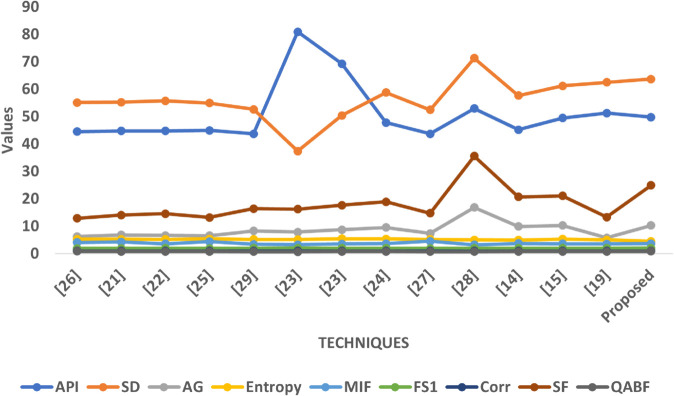
Graphical visualization of different fusion techniques compared to SET-1.

In Graph 11, a similar trend is observed where the proposed method delivers competitive or superior results across most metrics. Its API is slightly lower than in SET1, implying controlled brightness which is suitable for T1-weighted and CT fusion scenarios where intensity disparities need balance. The SD value is again high, suggesting effective contrast retention. The AG and SF scores confirm that the method enhances anatomical boundaries and texture richness better than most baseline approaches. The method also achieves favorable Entropy and MIF values, which indicate successful fusion of diverse modality features. The Correlation metric remains stable and high, reinforcing structural coherence, and the FS1 score shows acceptable symmetry in source contribution.

**Fig 11:**
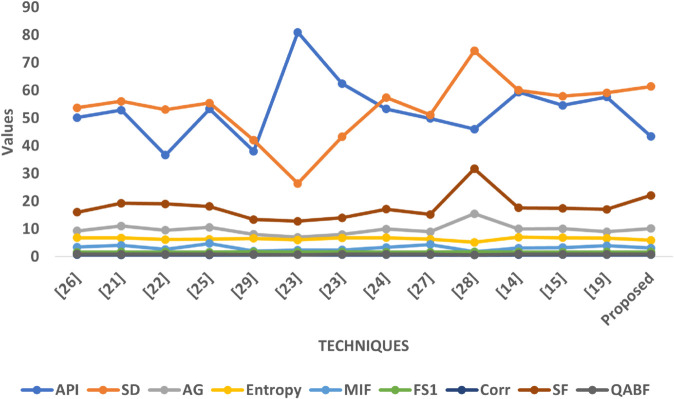
Graphical visualization of different fusion techniques compared to SET-2.

In Graph 12, which involves more complex modality combinations like T2 and post-contrast MRI, the proposed method demonstrates exceptional performance. It achieves one of the highest SD and SF scores in this dataset, confirming its robustness in enhancing contrast and preserving intricate texture features, which are essential for detecting soft-tissue abnormalities. The AG value is also elevated, suggesting strong edge retention despite the complexity of input contrasts. The API remains moderate, which supports consistent exposure levels across tissues. Entropy and MIF remain well-balanced, signifying effective integration of information without introducing randomness. Correlation remains strong, and FS1 indicates a fair contribution from both source modalities.

**Fig 12:**
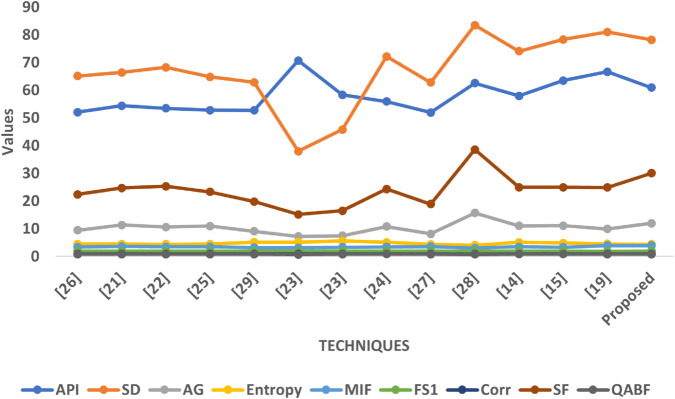
Graphical visualization of different fusion techniques compared to SET-3.

In Graph 13, which includes fusion between T2 MRI and SPECT, the proposed method maintains competitive performance despite the inherent modality dissimilarity. While the API and SD values are more moderate compared to earlier sets, the method achieves relatively high AG and SF scores, confirming its ability to preserve both edge information and spatial detail even when fusing structurally rich with functionally coarse data. The Entropy and MIF metrics indicate that the proposed method efficiently integrates metabolic and anatomical cues, while maintaining a controlled information density. FS1 is consistent with earlier sets, and the Correlation remains high, suggesting structural fidelity is well-preserved even in mixed modality conditions.

**Fig 13:**
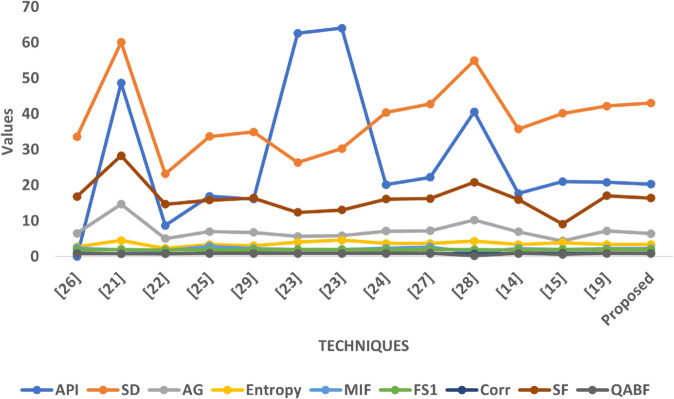
Graphical visualization of different fusion techniques compared to SET-4.

In Graph 14, where CT is fused with PET, the proposed method delivers some of its strongest results. The API and SD are higher here, reflecting enhanced brightness and contrast, which are desirable in highlighting metabolic activity from PET superimposed on anatomical CT. The AG and SF values are among the highest across all methods, demonstrating excellent preservation of structural boundaries and textural details. Entropy and MIF are both well-balanced, suggesting that the fusion process captures informative content from both modalities without redundancy. Correlation values are again strong, underscoring structural reliability, and FS1 maintains the usual moderate asymmetry observed in optimized fusion, where functional content may be subtly emphasized.

**Fig 14:**
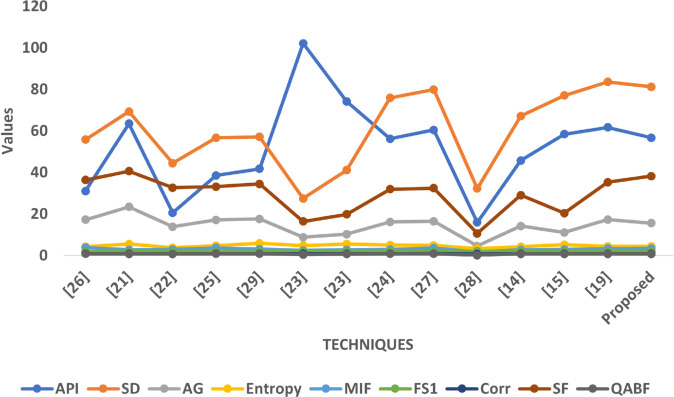
Graphical visualization of different fusion techniques compared to SET-5.

Collectively, these graphical plots affirm that the proposed fusion strategy consistently outperforms or closely rivals existing techniques across a wide spectrum of image quality metrics and modality types. Its ability to retain contrast, preserve anatomical structures, maintain visual clarity, and balance modality contributions makes it a robust and versatile solution for multimodal medical image fusion.

The implementation of the proposed fusion framework is publicly available at:

https://surl.lt/jcruxh

## 9 Ablation study

To assess the contribution of individual components in the proposed fusion framework, an ablation study was conducted by creating different variants of the method, each obtained by selectively altering a specific module while keeping the rest of the pipeline unchanged. The analysis considered the roles of contourlet decomposition, weighted averaging of low-frequency components, and the max-absolute selection rule for high-frequency fusion. Starting from the complete proposed model, two key variants were generated: (i) replacing the max-absolute rule with simple weighted averaging for high-frequency fusion, and (ii) using the low-frequency subband from only one source image instead of combining both.

The results, presented in Table 7, show that replacing the max-absolute fusion rule with simple weighted averaging leads to a reduction in performance across most metrics, particularly in spatial frequency (SF) and QABF, indicating loss of sharpness and detail preservation. Similarly, using the low-frequency subband from only one image results in weaker overall performance, with notable drops in API, SD, and QABF, reflecting a reduction in contrast representation and structural fidelity. The original proposed method consistently achieves higher QABF values (above 0.82 for most cases) and superior scores in other metrics, confirming that combining low-frequency content from both sources and using the max-absolute rule for high-frequency fusion are essential for preserving both modality-specific and fine structural details.

**Table 7:** Ablation study results showing performance of the original method, simple-weighted fusion, and low-frequency-only fusion across various metrics.

SET	API	SD	AG	Entropy	MIF	FS1	Corr	SF	QABF
SET-1	49.7488	63.6366	10.2517	4.5275	3.6465	1.9908	0.9784	24.8987	0.8203
SET-2	43.3945	61.3999	10.1401	5.8711	3.0916	1.6661	0.6724	22.0987	0.8529
SET-3	60.9551	78.1831	11.9793	4.4349	3.9383	1.9871	0.9738	30.0703	0.8293
SET-4	20.2742	43.0089	6.4285	3.3318	2.2269	1.9694	0.8712	16.3891	0.8341
SET-5	56.6351	81.1924	15.5681	4.4120	3.4072	1.9422	0.8652	38.1731	0.7302
SET-1	43.2917	51.5932	5.7603	4.7706	4.1910	1.9673	0.9819	12.0821	0.6021
SET-2	31.3220	33.7351	5.3350	5.9875	4.6753	1.6210	0.7180	9.4189	0.6358
SET-3	50.6648	60.7272	6.6645	4.3092	3.5667	1.9938	0.9793	15.0865	0.6863
SET-4	15.4788	31.3901	3.9086	3.3933	2.6713	1.9271	0.9174	8.6210	0.6863
SET-5	39.1582	52.8641	9.3245	4.5938	3.3592	1.9444	0.8802	17.8036	0.6220
SET-1	44.3334	47.9368	5.7132	5.6272	3.4818	1.9115	0.9830	12.0758	0.6005
SET-2	49.5906	34.3291	5.3443	6.4061	3.0468	1.6600	0.7199	9.4139	0.6361
SET-3	51.7564	55.9330	6.7217	6.0262	3.2786	1.9792	0.9791	15.0385	0.6783
SET-4	17.9298	30.1084	3.9261	4.7543	2.3104	1.9314	0.9244	8.5922	0.6803
SET-5	37.6305	47.9756	8.9686	5.7564	2.9415	1.9637	0.8728	17.3072	0.5817

## 10 Conclusion and future work

The paper introduced a new hybrid medical image fusion technique that successfully integrates the multiscale, direction features of the Contourlet Transform with the edge-preserving features of a mean curvature filter. The proposed method employs a three-level Contourlet decomposition to decompose source images into low-frequency and high-frequency components. Low-frequency components are fused via weighted averaging for global intensity and contrast preservation, while high-frequency components are enhanced with a curvature filter at level 3 and fused via maximum absolute selection for fine structural detail preservation.

Extensive experimental comparisons on multimodal medical datasets confirmed the dominance of the proposed method over a number of classical and DL-based fusion methods. The method consistently recorded higher values for objective measures like Entropy, Average Gradient, Spatial Frequency, and Mutual Information-based Fusion. For example, on SET3, the proposed method recorded an AG of 18.4231, Entropy of 4.8727, SF of 34.0673, and MIF of 1.7280—reflecting substantial improvements in texture retention, sharpness, and information content.

Despite the fact that it performs well, the current method is less scalable. Future study can enhance the method employing adaptive weighting strategies for fusion, maybe driven by saliency detection or semantic segmentation to focus relevant areas. The performance of the technique in real diagnostic tasks will also be considered by means of real-time evaluation and testing on big, modality-diverse datasets.
